# A microRNA signature for the differential diagnosis of salivary gland tumors

**DOI:** 10.1371/journal.pone.0210968

**Published:** 2019-01-25

**Authors:** Maria Denaro, Elena Navari, Clara Ugolini, Veronica Seccia, Valentina Donati, Augusto Pietro Casani, Fulvio Basolo

**Affiliations:** 1 Department of Surgical Pathology, Medical, Molecular and Critical Area, University of Pisa, Pisa, Italy; 2 Otorhinolaryngology Unit, Department of Surgical Pathology, Medical, Molecular and Critical Area, Azienda Ospedaliero-Universitaria Pisana, Pisa, Italy; 3 Department of Laboratory Medicine, section of Pathology Azienda Ospedaliero-Universitaria Pisana, Pisa, Italy; 4 Unit of Anatomic Pathology II, Azienda Ospedaliero-Universitaria Pisana, Pisa, Italy; University of Kansas School of Medicine, UNITED STATES

## Abstract

Salivary gland tumors (SGTs) are rare tumors of the head and neck with different clinical behavior. Preoperative diagnosis, based on instrumental and cytologic examinations, is crucial for their correct management. The identification of molecular markers might improve the accuracy of pre-surgical diagnosis helping to plan the proper treatment especially when a definitive diagnosis based only on cytomorphology cannot be achieved. miRNAs appear to be new promising biomarkers in the diagnosis and prognosis of cancer. Studies concerning the useful of miRNA expression in clinical decision-making regarding SGTs remain limited and controversial.The expression of a panel of 798 miRNAs was investigated using Nanostring technology in 14 patients with malignant SGTs (6 mucoepidermoid carcinomas, 4 adenoid cystic carcinomas, 1 acinic cell carcinoma, 1 ductal carcinoma, 1 cystadenocarcinoma and 1 adenocarcinoma) and in 10 patients with benign SGTs (pleomorphic adenomas). The DNA Intelligent Analysis (DIANA)-miRPath v3.0 software was used to determinate the miRNA regulatory roles and to identify the controlled significant Kyoto Encyclopedia of Genes and Genomes (KEGG) molecular pathways. Forty six miRNAs were differentially expressed (False Discovery Rate—FDR<0.05) between malignant and benign SGTs. DIANA miRPath software revealed enriched pathways involved in cancer processes as well as tumorigenesis, cell proliferation, cell growth and survival, tumor suppressor expression, angiogenesis and tumor progression. Interestingly, clustering analysis showed that this signature of 46 miRNAs is able to differentiate the two analyzed groups. We found a correlation between histological diagnosis (benign or malignant) and miRNA expression profile.The molecular signature identified in this study might become an important preoperative diagnostic tool.

## Introduction

Salivary gland tumors (SGTs) are rare and heterogeneous neoplasms of the head and neck. The preoperative management includes various instrumental and cytologic examinations that can be misleading for several reasons such as the many subtypes, the intratumoral heterogeneity and the morphological overlap patterns of these tumors[1].

Malignant lesions such as adenoid cystic carcinoma[2] or mucoepidermoid carcinoma[3] may be confused on cytology with Pleomorphic Adenoma (PA) because of the considerable overlap of the morphological patterns. The rate of false-negative results and poor accuracy for distinguishing between the various types of malignant SGTs are the main limitations of this technique. SGTs include rare lesions making difficult to acquire diagnostic expertise in Fine Needle Aspiration Cytology (FNAC)[4].

At the same time, for clinical-decision making it is important to determine whether a neoplasm is benign or malignant, because it guides the extent of the surgery. An improper preoperative diagnosis can lead to undertreat or overtreat a lesion worsening the outcome of the patient. The poor accuracy is due to the lack of a unique methods able to identify the features of SGTs. Although the diagnostic pitfalls are seen in a minority of cases, ancillary diagnostic markers are needed in order to overcome these cytological limitations and plan the proper treatment for such neoplasms.

microRNAs (miRNAs) are described as having roles in many diseases, including cardiovascular diseases[5], diabetes[6], neurodegenerative diseases[7,8], kidney diseases[9], and obesity[10]. miRNAs are also frequently deregulated in cancer. The deregulation of miRNAs (up- or down-regulation) affects the level of expression or the activity of tumor suppressors, oncogenes and other signaling molecules, and can cause DNA repair deficiencies, with a role in the development of human cancer [11]. According to the most recent evidences of the literature, miRNAs appear to be new promising biomarkers for diagnosis and prognosis of cancer[11–14].

To date, few studies have been published regarding the expression of miRNAs in SGTs. Some investigated the role of miRNAs in the progression of this type of cancer, particularly in two histotypes of SGTs: mucoepidermoid carcinoma and adenoid cystic carcinoma.

Binmadi et al. demonstrated the role of miR-302a in the invasion and aggressiveness of mucoepidermoid carcinoma[15]. Mitani et al. showed that deregulation of the miR-17-92 cluster may play a role in the biology of adenoid cystic carcinoma and might be a potential target for future therapeutic studies[16]. Chen et al. also analyzed miRNA profile in adenoid cystic carcinoma cells during metastatic progression, demonstrating up-regulation of miR-4487, -4430 and -486-3p, and down-regulation of miR-5191, -3131 and -211-3p[17].

Some authors studied miRNA expression in saliva samples that are more available and are easy to obtain. However, conflicting results have been obtained[18,19]. Independent studies reported deregulation of miRNAs in SGTs and differential miRNA expression profile between malignant, benign SGTs and normal tissue, increasing interest in the study of miRNAs in these neoplasms[19,20].

Therefore, in this study we investigated the expression of a wide panel of miRNAs (798 miRNAs) in patients with malignant SGTs and in patients with benign SGTs using an ultra-sensitive method: Nanostring technology. The aim of this study was to identify a miRNA signature for differential diagnosis between benign and malignant SGTs to use when a definitive diagnosis cannot be made on cytomorphology alone. This signature could improve diagnosis and allow better clinical decision-making with more appropriate treatment of SGTs.

## Materials and methods

Twenty-four patients who underwent parotidectomy from 2010 to 2015 at the Department of Surgical, Medical, Molecular and Critical Area Pathology, ENT Unit, University of Pisa, Italy were included in this study. Hematoxylin-eosin stained sections of patients were evaluated independently by two pathologists (C.U. and F.B.). A diagnostic concordance rate of 100% was achieved between the two investigators. The histopathological classification was performed in accordance with the WHO classification[21].

Among the 24 patients, 14 had malignant SGT, and 10 had PA. The latter is known to be the most frequent histological type among the benign neoplasms of the salivary gland.

The majority of malignant SGTs were mucoepidermoid carcinomas (42,9%, 6 out of 14 malignant SGTs) and adenoid cystic carcinomas (28,7%, 4 out of 14 malignant SGTs). The remaining malignant SGTs were classified as acinic cell carcinoma (7,1%, 1 out 14 malignant SGTs), ductal carcinoma (7,1%, 1 out 14 malignant SGTs), cystadenocarcinoma (7,1%, 1 out 14 malignant SGTs), and adenocarcinoma (7,1%, 1 out 14 malignant SGTs). Benign samples included 5 women and 5 men, while malignant samples included 8 women and 6 men. The mean age was 52 years (38–75) and 63 years (26–84) for the benign and the malignant tumor groups, respectively. Both groups consisted of the same ethnic background (Caucasian). Patient characteristics and tumor classification are summarized in Table 1.

**Table 1:** Patient characteristics included in the study.

	**Benign** **SGTs**	**Malignant** **SGTs**
**Mean age** (range)	52 (38–75)	63 (26–84)
**Sex** (M/F)	5/5	6/8
**Tumor subtypes**		
Pleomorphic adenoma	10	
Acinic cell carcinoma		1
Mucoepidermoid carcinoma		6
Adenoid cystic carcinoma		4
Ductal carcinoma		1
Cystoadenocarcinoma		1
Adenocarcinoma		1
**Grade**		
Low		6
High		8

All subjects gave their written informed consent for inclusion before they participated in the study. The study was conducted in accordance with the Declaration of Helsinki, and the protocol was approved by the Ethics Committee of Azienda Ospedaliera Universitaria Pisana, Comitato per la Sperimentazione Clinica dei Farmaci (Project identification code: 29937).

### miRNAs purification

For each sample, four 5-μm unstained sections were used for manual micro-dissection in order to increase the purified total RNA, including small RNAs. miRNAs were purified using the miRNeasy FFPE Kit (Qiagen Inc, Hilden, Germany) according to the manufacturer’s protocol. miRNA was eluted in 20 μL of RNase-free water. RNA quantification was assessed with a NanoDrop Spectrophotometer (Thermo Fisher Scientific, Waltham, Massachusetts, USA).

### nCounter human miRNA expression assay

The nCounter human v3 miRNA expression assay designed by Nanostring Technology (Nanostring, Seattle, WA, USA) was used in this study. This assay provided expression analysis of 798 human miRNAs using a pair of probes: a capture probe and a reporter probe. Furthermore, the assay included 5 housekeeping mRNAs (ACTB, B2M, RPL19 and RPLP0) for reference and control RNA to monitor the ligation efficiency and specificity between probes and target molecules.

For the analysis we used approximately 150 ng of purified total RNA as input material. According to NanoString recommendations, a 260/280 ratio of 1.9 or greater and a 260/230 ratio of 1.8 or greater were necessary to obtain optimal results.

### NanoString data normalization

The data produced by the nCounter Digital Analyzer contained the counts for each miRNA in a sample. Raw data of each miRNA were subjected to technical and biological normalization, using nSolver Software, version 2.5 (NanoString Technologies, Seattle, Washington).

Technical normalization allowed control of the variability unrelated to samples. If the calculated positive control scaling factor was outside a range of 0.3–3, it indicated technical problems, suggesting exclusion of the sample from further analysis.

Biological normalization, on the other hand, corrected for differences in RNA input among the assays. For each sample a biological normalization factor was determined. Whenever it was outside the range of 0.1–10.0, the sample was excluded from the analysis. miRNA input levels were normalized using the geometric mean of the top 100 miRNAs with the lowest variability coefficients. All the normalization steps were performed according to the manufacturers’ instructions (NanoString Technologies, Seattle, Washington).

### Functional analysis of miRNAs using DIANA-miRPath software

DNA Intelligent Analysis (DIANA)-miRPath v3.0 software (Vlachos et al. 2015) was used in order to identify the putative deregulated pathways by up- and down-regulated miRNAs between benign and malignant SGTs. This software links miRNAs to target genes from Tarbase, v7.0 and identifies the targeted ‘KEGG’ pathways. We used the ‘pathways union’ option of the miRPath software. *P*-values were obtained by the Fisher’s exact test as enrichment analysis method and the false discovery rate (FDR) was estimated using the Benjamini and Hochberg method[22].

### Statistical analysis

The differential miRNA expression between pleomorphic adenomas and malignant SGTs was determined by applying the non-parametric Mann–Whitney U-test followed by the Benjamini-Hochberg (BH) correction. The Mann–Whitney U test was carried out using IBM SPSS software package, version 17.0.1. The R ‘stats’ package, version 3.4.0 was used for the BH correction. A cut-off of 0.05 for FDR was used to consider a miRNA significant.

The clustering analysis was performed with nSolver Analysis software 2.5 using Pearson’s correlation.

The pathway analysis was carried out by DIANA-miRPath v3.0 software using FDR <0.05 as significant threshold[23].

## Results

In this study we analyzed the expression of 798 miRNA from 14 malignant SGTs, consisting of low and high-grade tumors, and from 10 PAs, using NanoString technology. Two out of 10 PAs were excluded for further analysis on the basis of the biological normalization factor.

To subtract the background noise, miRNAs with expression of less than two standard deviations from the mean of negative controls were not included in the analysis. This threshold allowed us to obtain 123 miRNAs suitable for analysis.

In the first step, we evaluated the expression profile of 22 samples using 123 miRNAs through unsupervised hierarchical clustering analysis, based on Pearson correlation. Subsequently, the same analysis was applied to 22 samples using miRNAs significantly deregulated between malignant and benign lesions.

### miRNA expression profile of malignant SGTs and pleomorphic adenomas

To evaluate the miRNA expression profile of analyzed malignant and benign tumors, we performed an unsupervised hierarchical clustering analysis using 123 miRNAs, with average count of two standard deviations (SD) from the mean of negative control (mean + 2 SD).

**Fig 1:**
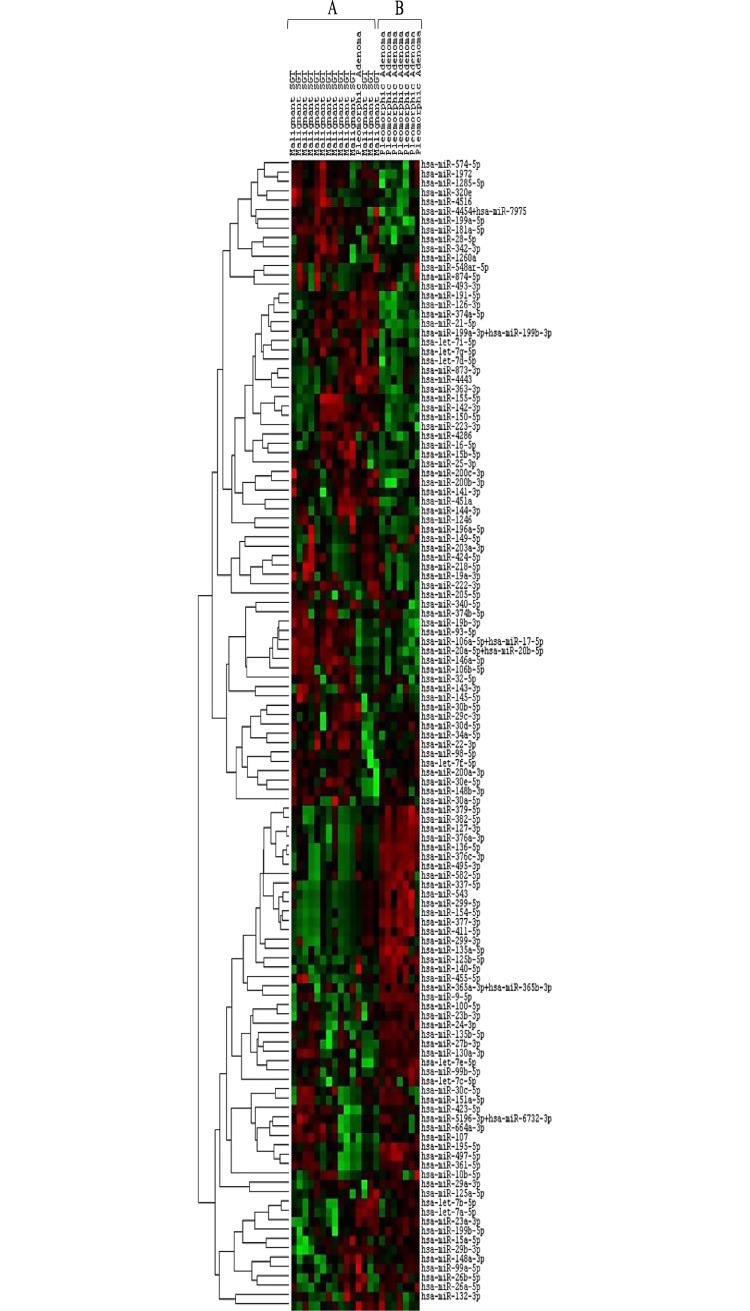
Unsupervised hierarchical clustering analysis of malignant SGTs and PAs using 123 miRNAs. The columns represent the cases, and the lines represent the miRNAs. Red color indicates high miRNA expression levels; green color indicates low miRNA expression level. Cluster A: 14 out of 14 malignant SGTs and one PA; cluster B: 7 out of 8 PAs.

Interestingly, we could identify two main expression profiles, represented by cluster A and cluster B. In detail, cluster A included all the malignant SGTs and only one PA, whereas cluster B included 7 out of 8 PAs (Fig 1).

### Differentially expressed miRNAs in malignant SGTs compared to pleomorphic adenomas

We performed a statistical analysis by using Mann-Whitney U-test in order to identify miRNAs that were differentially expressed between malignant and benign lesions.

We identified 61 differentially expressed miRNAs (*P*-value < 0.05) in malignant salivary gland tumors compared to PAs. Thirty-two out of 61 miRNAs were up-regulated, whereas 29 were down-regulated in malignant SGTs versus PAs. Applying the Benjamini-Hochberg correction, 46 out of 61 miRNAs had significant adjusted *P*-values (FDR < 0.05). Statistically significant miRNAs with *P*-values and adjusted *P*-values are summarized in Table 2.

**Table 2:** Significantly differentially expressed miRNAs between malignant SGTs and PAs.

**Upregulated miRNAs** **malignant SGTs vs PAs**	***P*-value**^**a**^	**Adjusted** ***P*-value**^**b**^
**hsa-miR-21-5p**	**0.0002**	**0.0031**
**hsa-miR-181a-5p**	**0.0003**	**0.0036**
**hsa-miR-199a-5p**	**0.0006**	**0.0063**
**hsa-miR-20a-5p+miR-20b-5p**	**0.0012**	**0.0104**
**hsa-miR-93-5p**	**0.0015**	**0.0116**
**hsa-miR-4286**	**0.0037**	**0.0229**
**hsa-let-7g-5p**	**0.0057**	**0.0260**
**hsa-miR-106a-5p+miR-17-5p**	**0.0057**	**0.0260**
**hsa-miR-199a-3p+miR-199b-3p**	**0.0057**	**0.0260**
**hsa-miR-106b-5p**	**0.0086**	**0.0320**
**hsa-miR-126-3p**	**0.0086**	**0.0320**
**hsa-miR-142-3p**	**0.0086**	**0.0320**
**hsa-miR-320e**	**0.0094**	**0.0340**
**hsa-miR-19b-3p**	**0.0105**	**0.0358**
**hsa-miR-200b-3p**	**0.0105**	**0.0358**
**hsa-miR-222-3p**	**0.0115**	**0.0373**
**hsa-miR-146a-5p**	**0.0127**	**0.0391**
**hsa-miR-374a-5p**	**0.0127**	**0.0391**
**hsa-miR-1246**	**0.0134**	**0.0402**
**hsa-miR-150-5p**	**0.0154**	**0.0412**
**hsa-miR-15b-5p**	**0.0154**	**0.0412**
hsa-miR-1285-5p	0.0222	0.0536
hsa-miR-4516	0.0222	0.0536
hsa-miR-191-5p	0.0222	0.0536
hsa-miR-451a	0.0222	0.0536
hsa-miR-1972	0.0265	0.0628
hsa-miR-342-3p	0.0316	0.0719
hsa-let-7d-5p	0.0374	0.0806
hsa-miR-144-3p	0.0441	0.0907
hsa-miR-4454+miR-7975	0.0441	0.0907
hsa-miR-155-5p	0.0442	0.0907
hsa-miR-19a-3p	0.0475	0.0957
**Downregulated miRNAs** **malignant SGTs vs PAs**	***P*-value**^**a**^	**Adjusted** ***P*-value**^**b**^
**hsa-miR-376c-3p**	**0.0002**	**0.0031**
**hsa-miR-125b-5p**	**0.0002**	**0.0031**
**hsa-miR-140-5p**	**0.0002**	**0.0031**
**hsa-miR-376a-3p**	**0.0002**	**0.0031**
**hsa-miR-127-3p**	**0.0002**	**0.0031**
**hsa-miR-136-5p**	**0.0002**	**0.0031**
**hsa-miR-495-3p**	**0.0002**	**0.0031**
**hsa-let-7e-5p**	**0.0004**	**0.0054**
**hsa-miR-377-3p**	**0.0007**	**0.0072**
**hsa-miR-99b-5p**	**0.0009**	**0.0088**
**hsa-miR-100-5p**	**0.0015**	**0.0116**
**hsa-miR-382-5p**	**0.0024**	**0.0172**
**hsa-miR-195-5p**	**0.0030**	**0.0193**
**hsa-miR-582-5p**	**0.0030**	**0.0193**
**hsa-miR-411-5p**	**0.0046**	**0.0258**
**hsa-miR-379-5p**	**0.0046**	**0.0258**
**hsa-miR-154-5p**	**0.0051**	**0.0260**
**hsa-miR-543**	**0.0051**	**0.0260**
**hsa-miR-135a-5p**	**0.0070**	**0.0288**
**hsa-miR-27b-3p**	**0.0070**	**0.0288**
**hsa-miR-9-5p**	**0.0070**	**0.0288**
**hsa-miR-337-5p**	**0.0113**	**0.0373**
**hsa-miR-299-5p**	**0.0137**	**0.0402**
**hsa-miR-135b-5p**	**0.0154**	**0.0412**
**hsa-miR-99a-5p**	**0.0154**	**0.0412**
hsa-miR-497-5p	0.0222	0.0536
hsa-miR-23b-3p	0.0316	0.0719
hsa-miR-199b-5p	0.0374	0.0806
hsa-miR-23a-3p	0.0374	0.0806

^a^*P*-values were obtained by using Mann-Whitney U test

^b^Adjusted *P*-values were obtained by using BH correction

**Fig 2:**
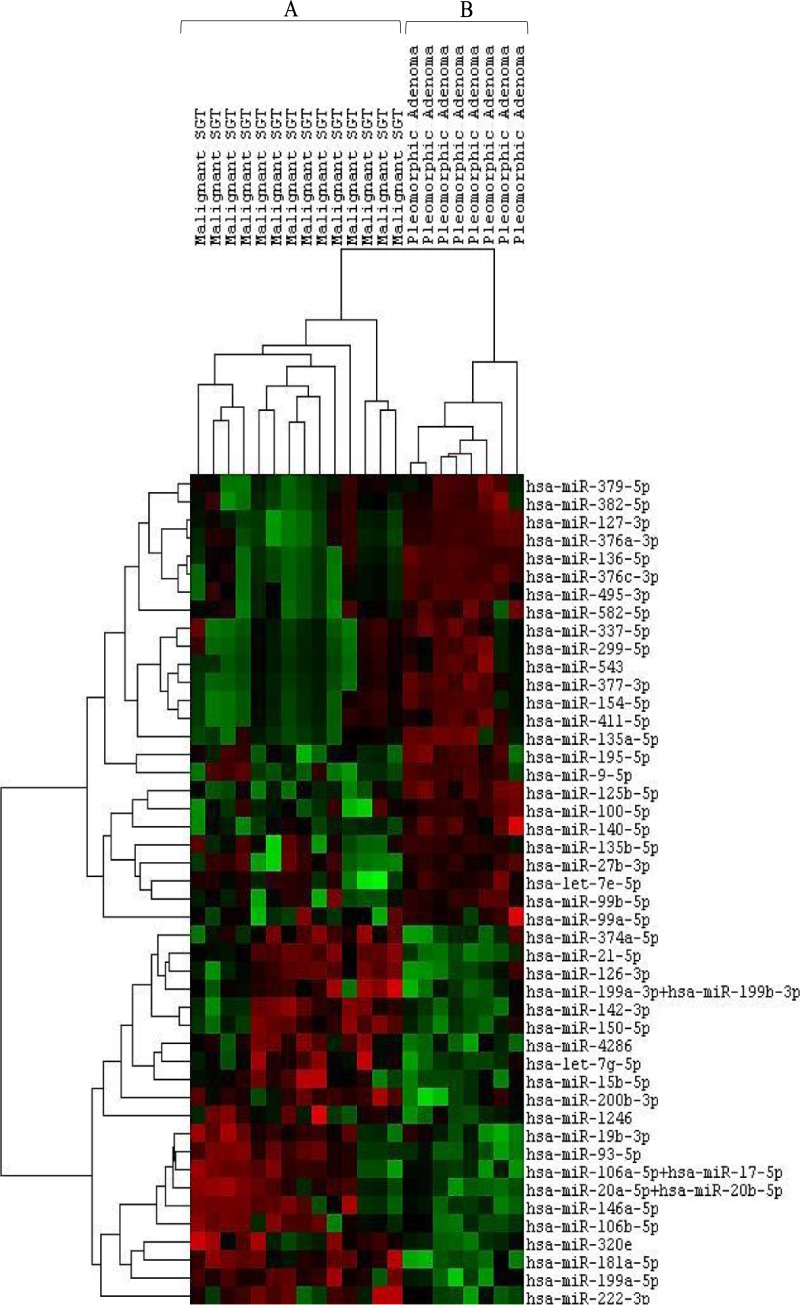
Hierarchical clustering analysis of malignant SGTs and PAs using differentially expressed miRNAs (FDR<0.05). The columns represent the cases, and the lines represent the miRNAs. Red color indicates high miRNA expression levels; green color indicates low miRNA expression level. Cluster A: 14 out of 14 (100%) malignant SGTs; cluster B: 8 out of 8 (100%) PAs.

In addition, we performed an unsupervised hierarchical clustering analysis using miRNAs with an adjusted *P*-value < 0.05. The signature of 46 miRNAs appeared to be histotype-specific. This allowed us to identify two clusters composed of malignant SGTs (cluster A) and benign SGTs (cluster B) (Fig 2).

## Enriched pathways identified by DIANA-miRPath software

DIANA miRPath v3.0 software revealed 44 enriched pathways with adjusted *P*-values <0.05. Among these, we selected 15 pathways involved in processes cancer related. The results are reported in Table 3.

**Table 3:** Enriched pathways: results using the DIANA-mirPath v3.0 software.

KEGG pathway maps	Enriched pathways	Adjusted *P-*values^a^
**Metabolism**		
Lipid metabolism	Fatty acid biosynthesis (hsa00061)	<1x10^-325^
	Fatty acid metabolism (hsa01212)	<1x10^-325^
**Environmental Information Processing**		
Signaling molecules and interaction	ECM-receptor interaction (hsa04512)	<1x10^-325^
Signal transduction	TGF-beta signaling pathway (hsa04350)	<1x10^-325^
	Hippo signaling pathway (hsa04390)	<1x10^-325^
	FoxO signaling pathway (hsa04068)	4,88x10^-13^
	PI3K-Akt signaling pathway (hsa04151)	0,0006
	mTOR signaling pathway (hsa04150)	0,0008
**Cellular processes**		
Cell growth and death	p53 signaling pathway (hsa04115)	<1x10^-325^
	Cell cycle (hsa04110)	5,32x10^-13^
Cellular community	Adherens junction (hsa04520)	9,21x10^-15^
	Focal adhesion (hsa04510)	1,34x10^-6^
**Human Diseases**		
Cancers: overview	Pathways in cancer (hsa05200)	<1x10^-325^
	Proteoglycans in cancer (hsa05205)	<1x10^-325^
	Transcriptional misregulation in cancer (hsa05202)	0,019

^a^Adjusted *P-* values were obtained by using the Benjamini–Hochberg method.

According to the KEGG pathway maps, 2 pathways were lipid metabolism related and 6 were environmental information processing related (1 was involved in signaling molecules and interactions and 5 were involved in signal transduction). The pathways related to cellular processes are involved in cellular growth and death (p53 signaling pathway and cell cycle) and in cellular community (adherens junction and focal adhesion).

In addition, pathways involved in cancer overview, such as pathways in cancer, proteoglycans in cancer and transcriptional misregulation in cancer were found significantly enriched.

## Discussion

SGTs constitute 3% of all head and neck tumors[21]. They include a heterogeneous group of lesions with various grades of malignancy, various histological features and various clinical-biological behaviors. FNAC is widely used as a first-line technique for the diagnosis of salivary gland pathologies [24]. However, it has not gained universal acceptance because of the high rate of false-negative results and poor accuracy for distinguishing among the different types of SGTs[25]. A more reliable diagnostic tool, such as the molecular markers identified in the present study, could improve the accuracy of pre-surgical assessment especially when a definitive diagnosis cannot be achieved based on cytomorphology only.

Whereas dysregulation of miRNA expression has been extensively reported in various types of tumors, including lung[26], breast[27], colon[28], stomach[27], pancreas[27], liver[29], prostate[30], thyroid[31], ovarian[32] and cervical cancers [33], studies concerning miRNA expression in SGTs are limited.

Among salivary gland malignancies, adenoid cystic carcinoma is the most common histotype studied. It is characterized by local recurrence, distant metastasis, perineural invasion, and poor long-term survival. Some authors, investigating miRNA expression in the metastatic progression of this carcinoma, demonstrated not only the potential role of miRNA in the spread of cancer cells but also their potential role as therapeutic targets for this type of cancer[16,17,34].

The value of salivary miRNA expression in clinical decision-making as a non-invasive diagnostic tool in SGTs remains controversial.

Matse et al. found on saliva samples that a combination of 4 miRNAs (miR-132, miR-15b, miR-140, and miR-223) was able to discriminate patients with malignant SGT from those with benign SGT[18]. Another independent study reported no differentially expressed miRNA on saliva samples between patients with benign neoplasms, malignant neoplasms and control groups [19]but the same authors showed a potential application of these molecular markers in the differential diagnosis when investigations were carried out on samples such as blood (plasma/serum) and tissues[19].

In particular, they found a statistically significant up-regulation in malignant SGTs of miR-199a and miR-30e in serum and plasma samples, respectively. The up-regulation of miR-199a-5p was also confirmed in tissue samples with miR-21, miR-31, miR-146b, and miR-345, when malignant salivary gland tumors were compared to the benign tumors[19].

To understand the mechanisms involved in the molecular biology of SGTs, Santos et al. compared the expression profiles of miRNAs in benign *vs* malignant tumors. Only miR-9 exhibited significantly increased expression in benign tumors, whereas miR-195 showed significantly decreased expression in malignant tumors[20].

Taken together, these published studies differed with respect to reporting differentially expressed miRNAs in benign and malignant SGTs.

Therefore, in our study, we investigated the expression of a wide panel of 798 miRNAs using an extremely sensitive method (Nanostring technology) in patients with malignant SGTs and in patients with benign SGTs in order to identify potential molecular markers for differential diagnosis when cytological analysis is inconclusive.

We found 46 miRNAs differentially expressed between malignant SGTs and PAs. Clustering analysis revealed that this molecular signature was able to differentiate benign from malignant lesions demonstrating a correlation between histological diagnosis and miRNA expression profile.

miR-21, which was one of the most significant miRNA found in our study, is crucial for embryological development and morphogenesis of the submandibular gland[35]. However, according to Zhang et al., this miRNA also appears to be involved in tumorigenesis of salivary gland tumors[36]. Several studies reported miR-21 as one of the most oncogenic miRNAs. Many tumor suppressors have been identified and experimentally confirmed as target genes for this miRNA: *PDCD4* in colorectal cancer[37], *PTEN* in hepatocellular cancer[38], *p53*, *TGF-β* and mitochondrial apoptosis tumor suppressor genes in glioblastoma cells[39]. Moreover, miR-21 was included among the 7 consistently up-regulated miRNAs in head and neck squamous cell carcinomas (HNSCC)[17]. In addition to these 7 up-regulated miRNAs, 4 microRNAs (miR-100, miR-99a, miR-125b, miR-375) have also been reported to be consistently down-regulated in HNSCC[17]. Among these, miR-100, miR-99a and miR-125b were found to be down-regulated also in our study. In particular, miR-125b down-regulation correlated with endometrial cancer invasion by targeting the proto-oncogene *ERBB2*[40] and inducing proliferation, colony formation, migration and invasion of cutaneous squamous cell carcinoma cells[41]. Functional analysis also showed the tumor suppressive role of miR-125b by inhibiting matrix metallopeptidase 13 (MMP13)[41]. However, down-regulation not only of miR-125b but also of miR-100 appeared to have an important role in development and progression of oral cancer cells (OCCs)[42]. miR-100 is one of the members of miR-99 family together with miR-99a. Deregulation of miR-99 family members was associated with cell proliferation[17,43–45], cell migration[46] and apoptotic processes[44]. These evidences were confirmed by Chen et al., since the forced expression of miR-99 family members reduced cell proliferation, cell migration and increased apoptosis in HNSCC cell lines[46].

Furthermore, the results of our study reported that miR-135a-5p, miR-195-5p, miR-199a-5p, miR-222-3p and miR-320e were differentially expressed between benign and malignant SGTs. Similar results have been reported by Borrelli et al. where miR-135a-5p, miR-195-5p and miR-222-3p were differentially expressed between benign and malignant also in thyroid tumors. The same authors proposed to evaluate miR-222-3p, miR-199a-5p and miR-320e in the diagnosis of pre-surgical fine-needle aspiration of indeterminate thyroid nodules[14].

A fundamental aspect that should not be overlooked in tumors development and progression is their interaction with the microenvironment.

In our study, among significantly down-regulated miRNAs, miR-195 and miR-9 were reported to be involved in the interaction of tumor cells with the microenvironment [20,36,47].

Zhang et al. found that anti-miR-9 inhibitor suppressed invasion, metastasis and angiogenesis in neuroblastoma cell lines [48]. By contrast, Santos et al. recently showed a significant negative correlation between miR-9 expression and microvessel density in salivary gland tumors[20]. Since miR-9 is secreted by fibroblasts the higher expression of miR-9 in our PAs may be explained by the rich extracellular matrix found in these tumors. According to these findings, the deregulation of miR-9 may be involved in the cross-talk between cancer cells and stroma, as reported by Baroni et al 2016[49].

Two independent research groups reported the relationship between miR-195 overexpression and inhibition of angiogenesis both in hepatocellular carcinoma and salivary gland tumors[20,44]. In our study miR-195 was significantly up-regulated in malignant lesions so it could contribute to the pathogenesis of these tumors.

However, of particular interest is that an association between some of deregulated miRNAs detected in our study and salivary gland tumors has already been reported.

miR-17-92 cluster was associated with the aggressive behavior of adenoid cystic carcinomas.[16]. Four out of 21 up-regulated miRNAs in our study belong to miR-17-92 cluster: miR-93, miR-106a, miR-20a, miR-106b. Therefore, the deregulation of these miRNAs is probably involved not only in pathogenesis of malignant SGTs but may also play a role in the biology and in the outcome of these tumors.

We have also verified that some of the deregulated miRNAs in our study, such as miR-374a-5p, miR-222-3p, miR-15b-5p, let-7g-5p and miR-140-5p, were also differentially expressed on saliva samples between patients with malignant tumors and patients with benign tumors in Matse et al. study [18].

In more details, miR-374 and miR-140 were up- and down-regulated, respectively, both in our malignant tissue samples and in saliva samples of Matse’s study[18]. Let-7g, miR-222 and miR-15b-5p appeared up-regulated in our malignant tissue samples but were down-regulated in saliva samples of patients with malignant tumors. These controversial results may arise from two points: first, the two studies used different subtypes of salivary gland tumors; second, total miRNAs were not freely circulating in saliva but may have been enclosed in microvesicles, primarily exosomes[50,51].

Comparing our results with the other evidences of the literature, we found that the 46 deregulated miRNAs identified in this study may influence tumor suppressor expression, cell invasion, cell proliferation, cell migration, apoptosis and angiogenesis in SGTs. We confirmed the correlation between the deregulation of these miRNAs and cellular processes in cancer by using the software DIANA miRPath v.3.0. Overall, this analysis revealed 44 enriched pathways and we selected 15 pathways cancer related. In addition to pathways involved in cancer overview, as well as pathways in cancer, proteoglycans in cancer and transcriptional misregulation in cancer, the pathways related to interaction between cell-cell and tumor cells-microenvironment (ECM-receptor interaction, adherens junction, focal adhesion), cell growth and death (p53 signaling pathway and cell cycle), lipid metabolism and signaling transduction (TGF-beta signaling pathway, Hippo signaling pathway, FoxO signaling pathway, PI3K-Akt signaling pathway, mTOR signaling pathway) were significantly enriched in this analysis.

These results indicate that the deregulation of these miRNAs influence cellular processes cancer related such as tumorigenesis (fatty acid synthesis, p53 signaling pathway, PI3K-Akt signaling pathway, mTOR signaling pathway)[52–55], cell proliferation (lipid metabolism, FoxO signaling pathway, mTOR signaling pathway, TGF-beta signaling pathway, Hippo signaling pathway)[55–59], cell growth and survival (mTOR signaling pathway, FOXO signaling pathway)[55,57], tumor suppressor expression (TGF-beta signaling pathway)[58,60], angiogenesis (TGF-beta signaling pathway)[61], and tumor progression (ECM-receptor interaction, adherens junction, focal adhesion, fatty acid synthesis, TGF-beta signaling pathway)[52,58,62–64].

## Conclusions

In conclusion, the deregulation of 46 miRNAs identified in our study may influence different processes cancer related.

Although a major sample size, more homogenous groups of lesions in terms of histology, and a prospective analysis on biological fluids are needed to strengthen the accuracy of our results, we believe that this is a good starting point. The signature of 46 miRNAs identified in our study might be potentially useful in differential diagnosis of SGTs when a definitive decision cannot be achieved based on only cytological analysis. In these cases, an accurate diagnosis will allow better clinical decision making with more appropriate treatment.
